# The association between levels of serum homocysteine and chronic heart failure: A protocol for systematic review and meta-analysis

**DOI:** 10.1097/MD.0000000000024117

**Published:** 2021-02-05

**Authors:** Xi Wang, Fu Wang, Zhiquan Feng, Jun Cai, Jianbin Liu

**Affiliations:** a Wuwei People's Hospital; b Gulang County People's Hospital, Wuwei, Gansu Province, China.

**Keywords:** cardiac function, CHF, Hcy, meta-analysis, systematic review

## Abstract

**Background:**

Homocysteine (Hcy) is one of the main factors leading to arteriosclerosis, which is closely related to cardiovascular disease. Recent studies have found that serum Hcy levels are increased in patients with chronic heart failure (CHF), and it is speculated that Hcy may be a risk factor for CHF, but evidence-based medicine evidence is lacking. The aim of this study was to investigate the correlation between serum Hcy levels and CHF by means of systematic review.

**Methods:**

The databases of PubMed, Embase, The Cochrance Library, Web of Science, CNKI (China National Knowledge Infrastructure), VIP (China Science and Technology Journal Database), Wanfang and China Biology Medicine disc were searched by computer. In addition, Baidu Scholar and Google Scholar were manually searched to collect all case–control studies related to serum Hcy and CHF. The search time limit was from database establishment to November 2020. Two reviewers independently screened the literatures, extracted the data and evaluated the risk of bias of the included literatures.

**Results:**

In this study, we evaluated the correlation between serum Hcy levels and CHF by the levels of serum Hcy in CHF patients and non-CHF patients.

**Conclusions:**

This study will provide reliable evidence for the clinical value of serum Hcy in the field of CHF disease.

**OSF Registration number:**

DOI 10.17605/OSF.IO/QMPRC.

## 1 Introduction

Chronic heart failure (CHF) is a clinical syndrome caused by impaired ventricular filling and ejection capacity which are caused by different etiologies, resulting in reduced ventricular ejection function and failure to meet systemic metabolic needs.^[1,2]^ Heart failure is a common critical illness in clinical practice, which has become one of the important cardiovascular diseases in the 21st century because of its high incidence and mortality. The results of a survey of 20 cities in 10 provinces of China showed that the prevalence of CHF in people aged 35 to 74 years was 0.9% in 2000.^[3]^ Research data in developed countries showed that the prevalence of heart failure was about 2.0%,^[4]^ and the prevalence of heart failure increases with age, and the prevalence of heart failure can reach more than 10% in people over 70 years of age.^[5]^ Patients with CHF are often accompanied by symptoms such as dyspnea, fatigue or fluid retention, which affects the quality of life and also brings a heavy burden to the society.^[6]^

Hcy is an important intermediate product in methionine and cysteine metabolism, and hyperhomocysteinemia (HHcy) is diagnosed when human serum Hcy level is greater than 15 μmol/L. HHcy is considered a marker of cardiovascular disease^[7]^ and a risk factor for diseases such as breast cancer,^[8]^ Alzheimer's disease,^[9]^ and age-related macular degeneration.^[10]^Some studies have also found a positive correlation between serum Hcy and BNP, HHcy can increase the 5-year risk of death in patients with heart failure.^[11]^ Tekin^[12]^ found that in patients with heart failure, serum Hcy gradually increased with the severity of cardiac function, and HHcy could reflect the severity of heart failure and may be an important predictor of mid-term death in patients with heart failure. At present, there are few studies on the relationship between serum homocysteine levels and CHF, and there is a lack of relevant evidence-based basis. Therefore, this study systematically evaluated the published studies on the relationship between serum homocysteine levels and patients with CHF at home and abroad in order to provide evidence-based medical evidence for the clinical value of Hcy in the field of CHF disease.

## 2 Methods

### 2.1 Protocol register

This protocol of systematic review and meta-analysis will be drafted under the guidance of the preferred reporting items for systematic reviews and meta-analysis protocols (PRISMA-P). It will be registered in the open science framework (OSF) on December 5, 2020 (registration number: DOI 10.17605/OSF.IO/QMPRC).

### 2.2 Ethics

Since this is a protocol with no patient recruitment and personal information collection, the approval of the ethics committee is not required.

### 2.3 Eligibility criteria

#### 2.3.1 Types of studies

We will collect all case–control studies on the relationship between serum Hcy levels and CHF, regardless of blind method, publication status, region, but language is limited to Chinese and English.

#### 2.3.2 Patients

Patients with definite diagnosis of CHF, including nationality, race, age, gender, and course of disease. Healthy people were used as controls.

#### 2.3.3 Exposure factors

Serum Hcy levels.

#### 2.3.4 Outcome indicators

Correlation between serum Hcy levels and CHF.

### 2.4 Exclusion criteria

1. Repeatedly published papers;
2. Non-Chinese and English literatures;
3. Literatures with incomplete data or unusable data;
4. The patient was clinically diagnosed with a disease (such as hypothyroidism, malignant swelling Tumor, chronic anemia) that had a significant impact on the results of the study;
5. Patients with CHF are taking immunosuppressive agents, antiepileptic drugs, etc.

### 2.5 Retrieval strategies

CNKI, Wanfang Data, VIP, PubMed, Cochrane Library, Embase, Web of Science, and other databases were searched by computer, and the retrieval time was from the establishment of database to November 2020. Take “chronic heart failure,” “congestive heart failure,” “homocysteine” as the Chinese search terms and search in Chinese databases, including CNKI, Wanfang, VIP, China Biology Medicine disc; Take “Cardiac Failure,” “Heart Decompensation,” “L-Isomer Homocysteine” as the English terms and search terms and search in English databases, including PubMed, EMBASE, Web of Science, the Cochrane Library. In addition, manual retrieval was conducted on Baidu academic and Google academic, and all domestic and foreign literatures on the relationship between serum homocysteine and CHF were collected. Taking PubMed as an example, the retrieval strategy was shown in Table 1.

**Table 1:** Retrieval strategy in PubMed database.

Number	Search terms
#1	Heart Failure[MeSH]
#2	Cardiac Failure[Title/Abstract]
#3	Heart Decompensation[Title/Abstract] OR Decompensation, Heart[Title/Abstract]
#4	Heart Failure, Right-Sided[Title/Abstract] OR Heart Failure, Right Sided[Title/Abstract] OR Right-Sided Heart Failure[Title/Abstract] OR Right Sided Heart Failure[Title/Abstract]
#5	Myocardial Failure[Title/Abstract]
#6	Congestive Heart Failure[Title/Abstract] OR Heart Failure, Congestive[Title/Abstract]
#7	Heart Failure, Left-Sided[Title/Abstract] OR Heart Failure, Left Sided[Title/Abstract] OR Left-Sided Heart Failure[Title/Abstract] OR Left Sided Heart Failure[Title/Abstract] OR CHF
#8	#1 OR #2 OR #3 OR #4 OR #5 OR #6 OR #7
#9	Homocysteine[MeSH]
#10	2-amino-4-mercaptobutyric acid[Title/Abstract] OR 2 amino 4 mercaptobutyric acid[Title/Abstract]
#11	Homocysteine, L-Isomer[Title/Abstract] OR Homocysteine, L Isomer[Title/Abstract] OR L-Isomer Homocysteine OR Hcy
#12	#9 OR #10 OR #11
#13	#8 AND #112

### 2.6 Data filtering and extraction

Referring to the Methods for inclusion in the Cochrane Handbook of Systematic Reviewers (Version 5.0), according to the PRISMA flow chart, two researchers independently screened the literature using EndNote X7 literature management software based on the above inclusion and exclusion criteria. The third researcher discussed and determined those studies that were difficult to include due to differences. At the same time, Excel 2013 was used to extract relevant information, including:

1. Clinical research (title, first author, publication date, basic characteristics of the research object);
2. Exposure factors (serum Hcy levels in the control group; Serum Hcy levels of CHF group);
3. Key elements of bias risk assessment; Observation index.

**Figure 1:**
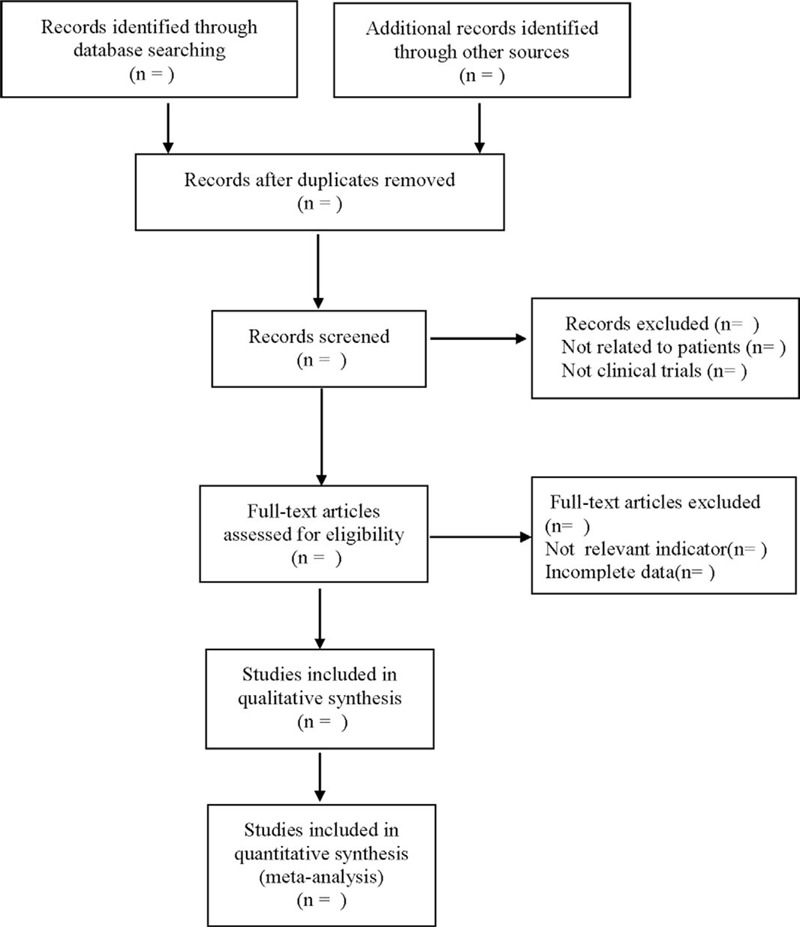
Flow diagram.

The literature selection process was shown in Figure 1.

### 2.7 Literature quality assessment

The risk of bias of the included studies was independently evaluated by two investigators and the results were cross-checked, and the risk of bias of case–control studies was evaluated using the Newcastle-Ottawa-Scale (NOS) scale.

### 2.8 Statistical analysis

RevMan5.3 software will be used for Meta analysis. For continuous weighted mean difference (WMD) is used if the measurement tool is consistent with the unit of measurement. If the measurement tool or unit of measurement is inconsistent, the standard mean difference (SMD) is used as the effect size. Each effect size is provided with its point estimate and 95% confidence interval (CI). The heterogeneity among the included study results was analyzed by χ^2^ test (the test level was α = 0.1), and the heterogeneity was quantitatively judged in combination with *I*^2^. If (*P *≥ .1, *I*^2^ ≤ 50%) indicated low heterogeneity, the fixed-effect model was used for Meta analysis. If (*P* < .01, *I*^2^ > 50%) indicates heterogeneity among studies, the source of heterogeneity shall be analyzed. Clinical heterogeneity was treated by subgroup analysis. Statistical heterogeneity was considered if there was no significant clinical heterogeneity and methodological heterogeneity, so the random effects model was used for analysis. If the clinical heterogeneity is too obvious to be analyzed, the meta-analysis will not be performed, but descriptive analysis will be performed.

#### 2.8.1 Dealing with missing data

If the article had missing data, the authors were contacted by email to supplement the relevant information. If the authors could not be contacted, or the authors had lost relevant data, descriptive analysis was performed instead of meta-analysis.

#### 2.8.2 Subgroup analysis

Take subgroup analysis according to the geographical region; take subgroup analysis according to age; take subgroup analysis according to the included sample size.

#### 2.8.3 Sensitivity analysis

To determine the stability of the outcome measures, sensitivity analysis was used to analyze each outcome indicator.

#### 2.8.4 Assessment of reporting biases

If the number of studies included in an outcome measure was not <10, a funnel plot was used for the assessment of publication bias. In addition, Egger's and Begg's test publication was used for the evaluation of potential bias.

#### 2.8.5 Evidence quality evaluation

The Grading of Recommendations Assessment, Development, and Evaluation (GRADE) will be used to assess the quality of evidence. It contains 5 domains (bias risk, consistency, directness, precision, and publication bias). And the quality of evidence will be rated as high, moderate, low, and very low.

## 3 Discussion

The occurrence of CHF is often accompanied by changes such as myocardial overload, myocardial cell injury, and apoptosis, which are mainly caused by injury factors such as coronary heart disease, hypertension, and inflammation, and finally lead to cardiac pumping dysfunction, which is a clinical manifestation of the development of cardiac disease to a serious and final stage.^[2,13]^ CHF is a progressive disease, and some studies suggested that the occurrence and development of CHF are associated with ventricular remodeling,^[14]^ changes in the sympathetic nervous system,^[15]^ endothelin,^[16]^ and Hcy,^[17]^ and its pathological and physiological mechanisms are more complex. In clinical practice, heart failure is diagnosed and evaluated mainly through medical history and relevant examinations. Comprehensive and accurate diagnosis is the premise and basis of effective treatment for patients with heart failure. Therefore, how to accurately diagnose and evaluate patients’ condition at an early stage has become the main research direction of many scholars.

Hcy is a sulfur-containing amino acid, mainly derived from dietary intake of methionine. Some studies have reported that serum homocysteine can promote the production of oxygen free radicals in the body, damage vascular endothelial cells and vascular function, and further cause intimal thickening and stiffness of blood vessels, leading to atherosclerosis.^[18]^Raaf^[19]^ and others have found that HHcy can increase the level of transforming growth factor-β1 (TGFβ1), affect the balance between TIMP1 and MMP1, lead to dysregulation of extracellular matrix degradation, cause extracellular matrix remodeling, and affect cardiac function. In addition, homocysteine also increases the expression of type I collagen in cardiac fibroblasts and cause myocardial extracellular matrix remodeling.^[20]^Therefore, HHcy will cause cardiomyocyte extracellular matrix remodeling, leading to myocardial tissue damage, with the changes in cardiac structure and infiltration of inflammatory cells, cardiac function gradually decreases, and finally heart failure develops.^[21]^Okuyan^[22]^ also confirmed that serum Hcy levels are generally increased in patients with CHF, suggesting that serum Hcy may be involved in the process of CHF development. Other studies^[23]^ have found that serum Hcy levels are related to the severity of heart failure patients, and also affect the prognosis of patients. In a study of 108 CHF patients, it was found that the 3-year survival rate was 37% in patients with HHcy, while the 3-year survival rate was 73% in patients with normal Hcy, considering that HHcy can reflect the severity of CHF patients and is related to poor prognosis, whether the treatment of HHcy can improve the prognosis of CHF remains to be further studied.^[24]^ Therefore, it is necessary to analyze the existing studies on the relationship between serum Hcy level and CHF, and objectively evaluate the value of Hcy in the diagnosis, severity and prognosis of CHF, so as to provide reliable evidence for clinicians to apply Hcy to CHF patients. This study also has some limitations, with few included articles and small sample size. As only case–control studies were included, implementation and measurement bias could not be avoided due to the limitations of study design, and the time relationship between the increase of serum Hcy levels and the occurrence and development of CHF could not be determined. Due to the limitation of language ability, we only search the English and Chinese literatures, and may ignore the studies or reports in other languages.

## Author contributions

**Data curation:** Xi Wang, Fu Wang.

**Funding acquisition:** Jianbin Liu.

**Investigation:** Fu Wang, Jun Cai.

**Software:** Zhiquan Feng, Jun Cai.

**Supervision:** Zhiquan Feng, Jun Cai.

**Writing – original draft:** Xi Wang, Fu Wang.

**Writing – review & editing:** Xi Wang, Jianbin Liu.
